# BNIP3L promotes cardiac fibrosis in cardiac fibroblasts through [Ca^2+^]_i_-TGF-β-Smad2/3 pathway

**DOI:** 10.1038/s41598-017-01936-5

**Published:** 2017-05-15

**Authors:** Weili Liu, Xinxing Wang, Zhusong Mei, Jingbo Gong, lishuang Huang, Xiujie Gao, Yun Zhao, Jing Ma, Lingjia Qian

**Affiliations:** 1 Tianjin Institute of Health and Environmental Medicine, No. 1 Da Li Road, Heping District, Tianjin, 300050 China; 2 Beijing Institute of Basic Medical Sciences, No. 27 Taiping Road, Haidian District, Beijing, 100850 China

## Abstract

Fibrosis is an important, structurally damaging event that occurs in pathological cardiac remodeling, leading to cardiac dysfunction. BNIP3L is up-regulated in pressure overload-induced heart failure and has been reported to play an important role in cardiomyocyte apoptosis; however, its involvement in cardiac fibroblasts (CFs) remains unknown. We prove for the first time that the expression of BNIP3L is significantly increased in the CFs of rats undergoing pressure overload-induced heart failure. Furthermore, this increased BNIP3L expression was confirmed in cultured neonatal rat CFs undergoing proliferation and extracellular matrix (ECM) protein over-expression that was induced by norepinephrine (NE). The overexpression or suppression of BNIP3L promoted or inhibited NE-induced proliferation and ECM expression in CFs, respectively. In addition, [Ca^2+^]_i_, transforming growth factor beta (TGF-β) and the nuclear accumulation of Smad2/3 were successively increased when BNIP3L was overexpressed and reduced when BNIP3L was inhibited. Furthermore, the down-regulation of TGF-β by TGF-β-siRNA attenuated the increase of BNIP3L-induced fibronectin expression. We also demonstrated that the increase of BNIP3L in CFs was regulated by NE-AR-PKC pathway *in vitro* and *in vivo*. These results reveal that BNIP3L is a novel mediator of pressure overload-induced cardiac fibrosis through the [Ca^2+^]_i_-TGF-β-Smad2/3 pathway in CFs.

## Introduction

Cardiac remodeling, including cardiomyocyte hypertrophy, apoptosis and cardiac fibrosis, adversely promotes left ventricular dilation and myocardial stiffness, resulting in advancing stages of heart failure^1, 2^. Thus, preventing the progression of cardiac remodeling is expected to suppress that of heart failure^3^. Moreover, there is evidence that increased fibrosis, rather than cardiac hypertrophy, may be the most significant cause of diastolic dysfunction in hypertrophic cardiac disease^4, 5^. Cardiac fibrosis is defined as the excessive production and abnormal accumulation of extracellular matrix (ECM) proteins, which can ultimately lead to cardiac structural damage and dysfunction^3, 6, 7^. Studies of cardiac fibrosis in CFs have flourished over the last decade. In the process of cardiac fibrosis, complex molecular mechanisms play critical roles in regulating cardiac fibroblast activation and ECM deposition^8, 9^. However, therapies based on these mechanisms still cannot effectively prevent cardiac fibrosis. A better understanding of the mechanisms for the functional decompensation of cardiac fibrosis is essential to the development of effective preventative measures and therapies^9, 10^.

BNIP3L/Nix is a nearly ubiquitous member of the Bcl-2 family that is expressed at very low levels in normal hearts but was originally described a s a transcriptionally up-regulated gene in hemodynamic overload and cardiac-specific Gq-overexpressing mice^11–14^. BNIP3L transcription was also found to be increased by phenylephrine but not by isoproterenol, angiotensin II, or hypoxia in cardiomyocytes. *In vivo*, BNIP3L was found to participate in the progression of heart failure in adult rats with TAC using specifically ablation in cardiomyocytes (KO) and conditionally overexpressed it in the heart^15–17^. The group of G. Dorn demonstrated that cardiomyocyte-specific BNIP3L knockout mice after TAC had less TUNEL positivity and caspase 3 and PARP cleavage. Also, late fibrotic replacement of dead cardiac myocytes was also reduced^17^. These results indicated apoptotic cell death of cardiomyocytes induced by BNIP3L indirectly leads to increased fibrosis, but the direct effect of BNIP3L on CFs remains unknown.

In the present study, the cellular distribution of BNIP3L during pressure overload -inducedcardiac remodeling was detected. We found that BNIP3L expression is not only upregulated in cardiac myocytes, but also significantly up-regulated in CFs. Subsequent studies have confirmed that BNIP3L expression increased in cultured neonatal CFs that was induced by NE. Moreover, BNIP3L was up-regulated by the NE-adrenoceptor (AR)-PKC pathway in CFs. To investigate the direct role and molecular mechanisms of BNIP3L in CFs, we used the transfection of plasmid DNA and siRNA of BNIP3L. Our results support a critical role for BNIP3L in CFs to regulate cell proliferation and ECM expression through the [Ca^2+^]_i_-TGF-β-Smad2/3 pathway. *In vitro* studies have indicated that intracellular Ca^2+^ signaling is an important second messenger of the TGF-β signal transduction pathway^18, 19^. A large body of evidence suggests that the development of cardiac fibrosis is controlled by a regulatory network involving TGF-β and Smad2/3^20, 21^. Together, these data suggest an important role and molecular mechanism for BNIP3L in CFs during pressure overload-induced heart failure.

## Results

### BNIP3L expression increases during the development of pressure overload-induced cardiac fibrosis

Hypertension is one of the most common causes of cardiac remodeling and dysfunction, which eventually leads to heart failure^22^. To explore the impact of pressure overload on the heart, a microsurgical approach was used to induce hypertension by pressure overload following AAC. Compared with the control and sham, the blood pressure of the AAC rats increased after 2 week and remained high for 8 weeks, indicating that these rats were subjected to long-term pressure overload (Supplementary Fig. S1a). Echocardiographic assessment showed that the left ventricular (LV) mass increased after 4 weeks of AAC and the ejection fraction and fractional shortening decreased after 6 weeks of AAC (Supplementary Fig. S2). Therefore, these results demonstrate that AAC-induced hypertension leads to heart failure.

**Figure 1:**
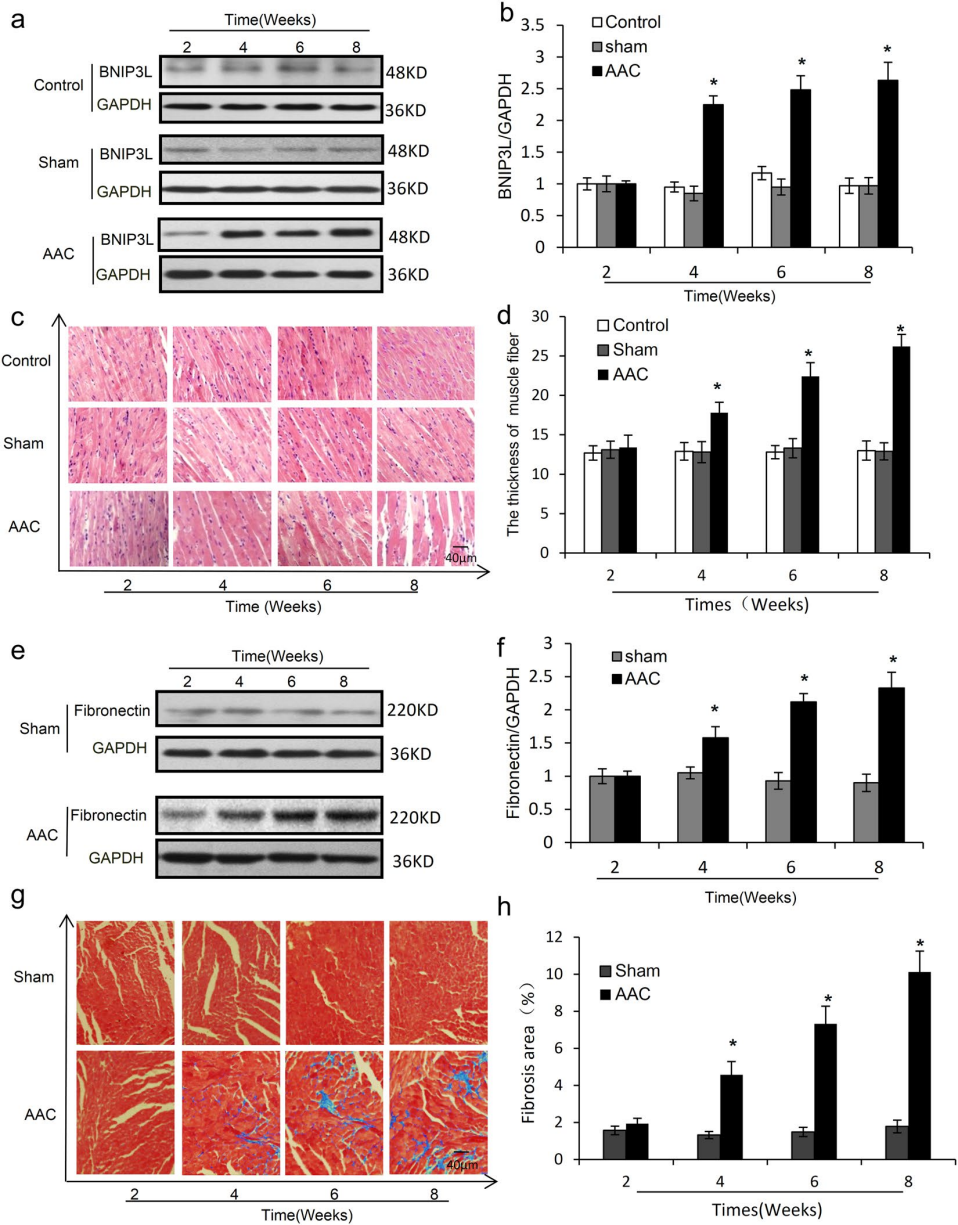
BNIP3L expression is consistent with myocardial hypertrophy and fibrosis *in vivo*. The rats were subjected to abdominal aortic constriction (AAC) surgery. (**a**) Western blot analysis showing the effects of pressure overload on BNIP3L expression. (**b**) Densitometric analysis of blots for determining BNIP3L normalized to GAPDH. (**c**) Representative histological images with hematoxylin and eosin staining of heart sections. (**d**) Quantification of muscle fiber thickness from hematoxylin and eosin staining of heart sections. (**e**) Western blot analysis showing the effects of pressure overload on fibronectin expression. (**f**) Densitometric analysis of blots for determining fibronectin normalized to GAPDH. (**g**) Masson’s trichrome staining showing the effects of pressure overload on fibrotic lesions. (**h**) Mean values of the ratio of collagen surface area to the myocardial surface area expressed as percentage of fibrosis. Data shown are mean ± SD, *P ≤ 0.05 vs. Control and sham, n = 8.

To explore the changes in BNIP3L expression during heart failure, we detected its expression levels by western blot and found that they were dramatically increased after 4 weeks of AAC (Fig. 1a,b). The heart weight to body weight ratios of the AAC rats increased after 4 weeks (Supplementary Fig. S1b). The gross morphology of the whole hearts of the AAC rats showed more pronounced myocardial enlargement (Supplementary Fig. S1c).The cross sectional area of cardiomyocytes were also increased after 4 weeks of AAC (Supplementary Fig. S1d). In addition, hematoxylin and eosin staining demonstrated that the muscle fibers were significantly thicker (Fig. 1c,d). Apoptosis and fibrosis have been shown to be major pathological events in the development of pressure overload-induced heart failure^23^. TUNEL assays revealed an increased number of TUNEL-positive cardiac myocytes in sections from rats following 6 and 8 weeks of AAC (Supplementary Fig. S3). Western blot analysis revealed that the expression of fibronectin, an important extracellular matrix (ECM) protein, was markedly increased in the AAC rats after 4 weeks (Fig. 1e,f). Furthermore, Masson’s trichrome staining of the heart sections demonstrated a marked increase in collagen accumulation in the AAC rats after 4 weeks (Fig. 1g,h). Therefore, these results demonstrate a clear relationship between BNIP3L expression and cardiac hypertrophy, apoptosis and cardiac fibrosis during pressure overload-induced heart failure.

### BNIP3L is located not only in cardiomyocytes but also in CFs

**Figure 2:**
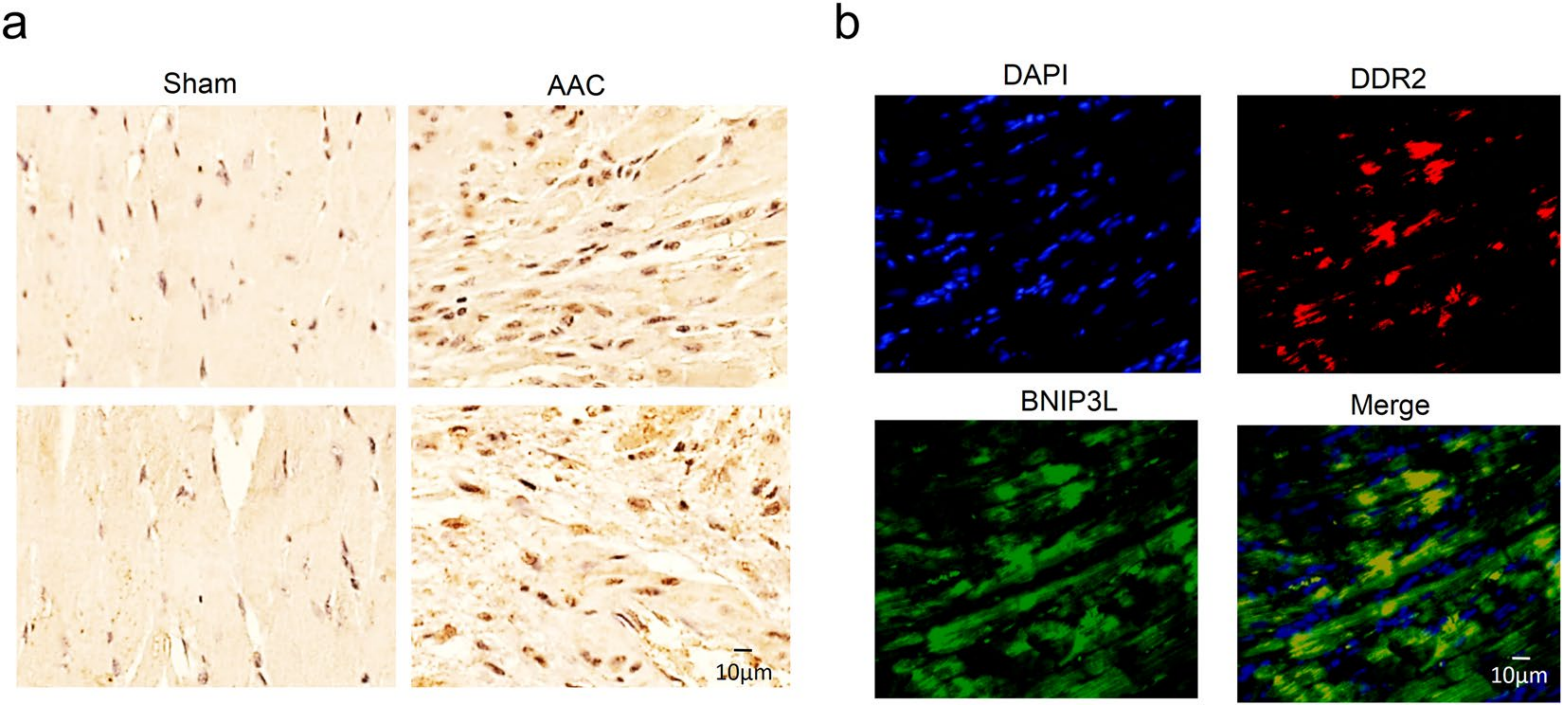
BNIP3L is up-regulated in CFs during pressure overload-induced cardiac remodeling. (**a**) Immunohistochemistry showing the cellular distribution of BNIP3L. (**b**) Immunofluorescence showing the co-localization of BNIP3L and DDR2.

Previous studies about the role of BNIP3L focused on cardiomyocyte apoptosis. To a better understanding of the function of BNIP3L in hypertensive heart disease, we detected the cellular distribution of BNIP3L in the heart sections by immunohistochemistry. Compared with the sham rat hearts, the number of cells in the interstitium of the AAC rats increased. Moreover, BNIP3L was not only up-regulated in cardiomyocytes but also significantly in interstitial cells (Fig. 2a). Furthermore, we determined the relationship between BNIP3L and CFs by immunofluorescence. We found that BNIP3L co-locates with DDR2, a cardiac fibroblast-specific protein (Fig. 2b), indicating that it is significantly up-regulated in the CFs during pressure overload-induced cardiac remodeling.

### BNIP3L expression increases in NE-induced CFs *in vitro*

**Figure 3:**
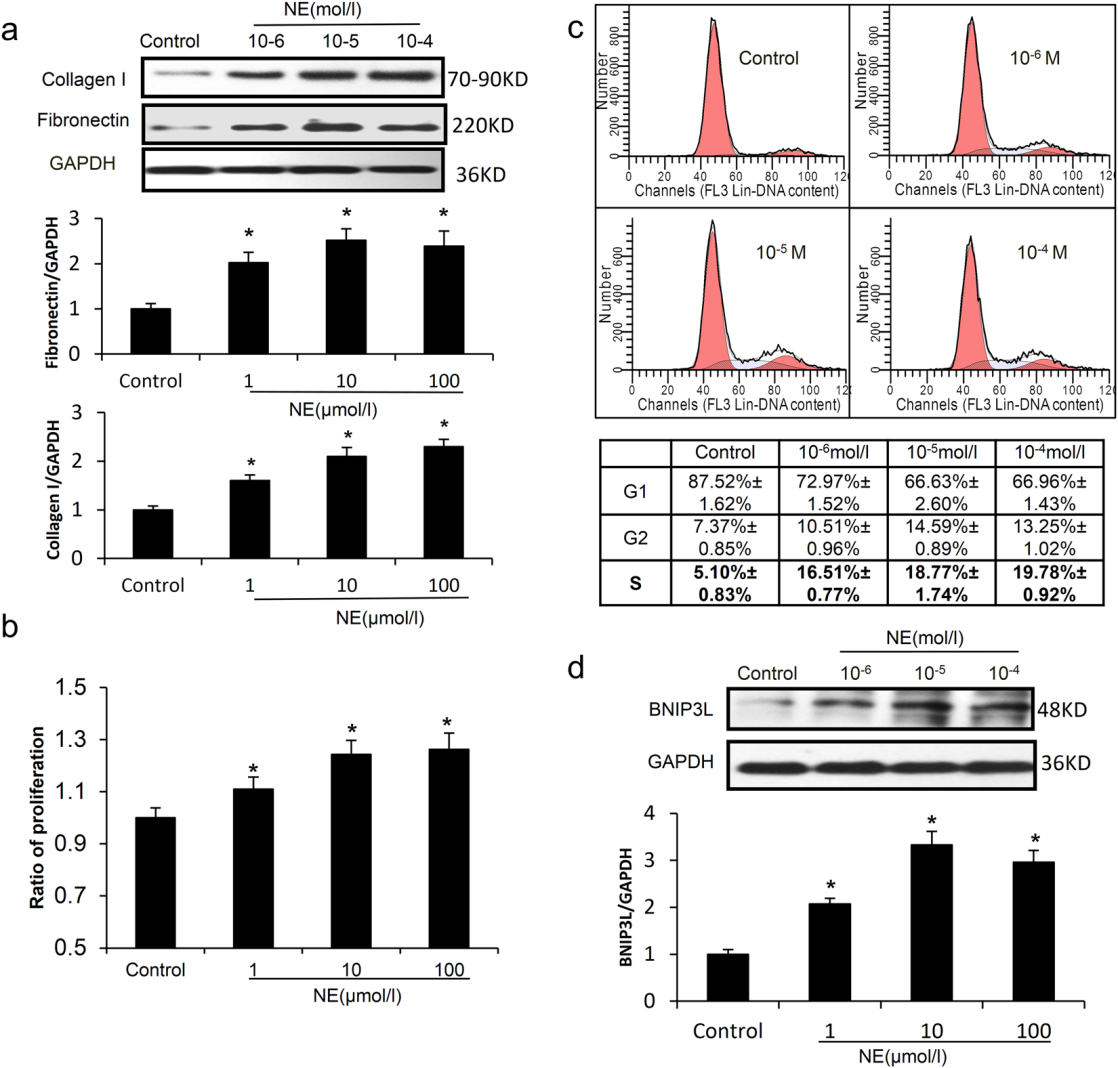
The effects of NE on cell fibrosis and BNIP3L expression in CFs. Neonatal rat CFs were treated with different concentrations of NE for the indicated times. (**a**) Western blot analysis showing NE-induced fibronectin and collagen I expression. (**b**) Proliferation was measured using the MTT assay. (**c**) Cells were stained with PI and examined by FACS. The data were analyzed using the ModFit program. (**d**) Western blot analysis showing NE- induced BNIP3L expression. Each experiment repeated three times. Data shown are mean ± SD, *P < 0.05 vs. Control.

To confirm the relationship between BNIP3L expression and fibrosis, CFs were isolated from the left ventricles of neonatal rats. During hypertension, the secretion of norepinephrine (NE) increased (Supplementary Fig. S4). To determine the effects of NE on fibrosis, the neonatal rat CFs were incubated with 10^−6^–10^−4^ mol/l NE for 24 hour or 10^−5^ mol/l NE for the indicated times. Flow cytometry analysis showed no significant increase of cell apoptosis in the NE-induced CFs (Supplementary Fig. S5). However, compared with the parental controls, the MTT assay showed a significant increase in the cell proliferation rate in a dose and time-dependent manner (Fig. 3b and Supplementary Fig. S6a). To identify a potential mechanism for this NE-induced cell proliferation, the cell cycle distribution was assessed using flow cytometry, which showed that the percentage of cells in the DNA synthesis phase (S phase) significantly increased (Fig. 3c and Supplementary Fig. S6b). Fibronectin and collagen, the important extracellular matrix (ECM) component, were also increased in the NE-induced CFs (Fig. 3a and Supplementary Fig. S6c). Furthermore, western blot showed that NE had a stimulatory effect on BNIP3L expression in CFs in a dose and time-dependent manner (Fig. 3d and Supplementary Fig. S6d). These results indicate that BNIP3L expression is closely related to CFs proliferation and ECM protein expression.

### BNIP3L promotes cell proliferation and ECM protein over-expression in CFs

**Figure 4:**
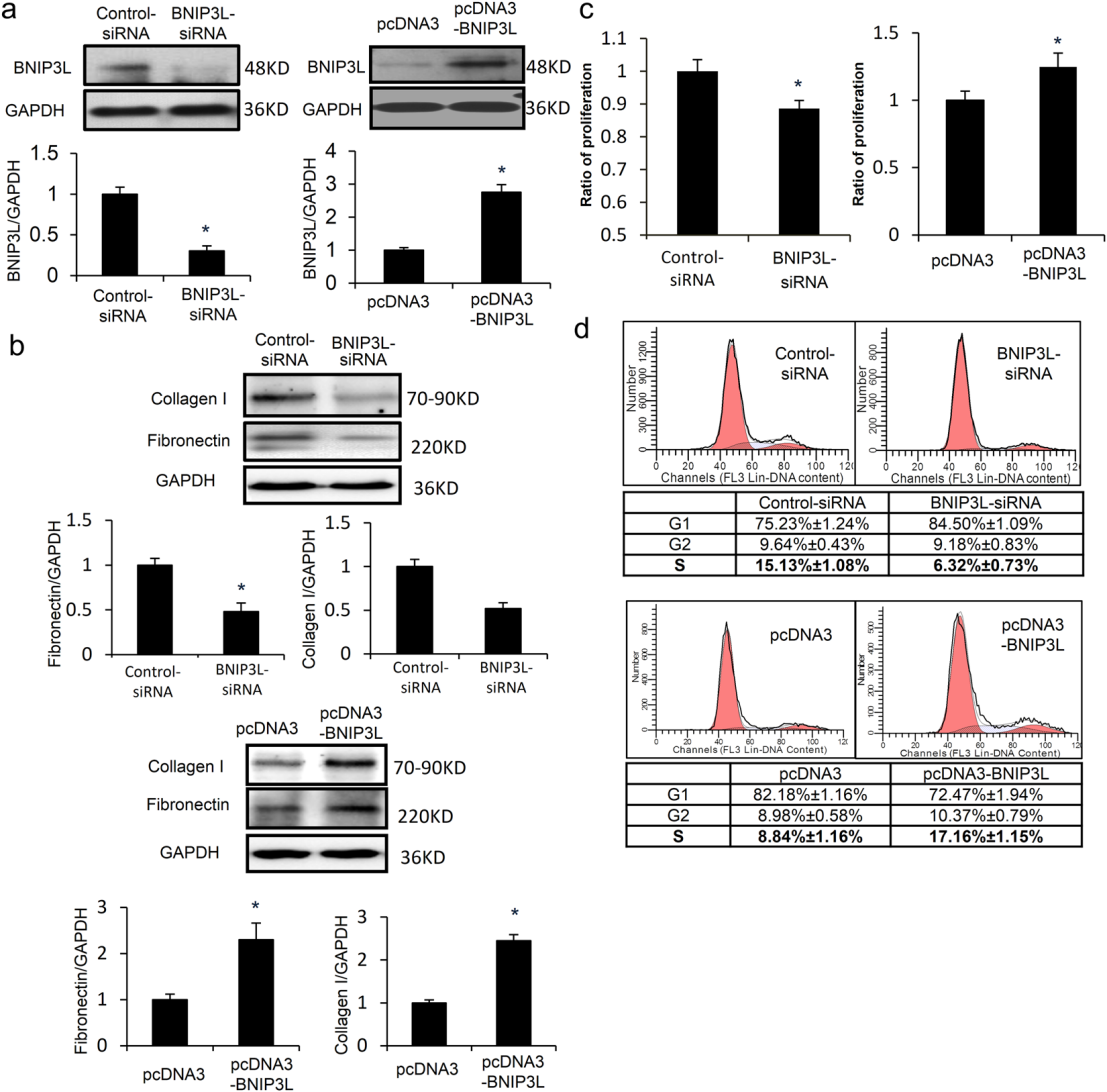
The roles of BNIP3L in CFs. Neonatal rat CFs were transfected with the BNIP3L and BNIP3L-siRNA adenoviruses. (**a**) Western blot analysis showing the effects of the adenoviruses on BNIP3L expression. (**b**) Western blot analysis showing the effects of BNIP3L on fibronectin and collagen I expression. (**c**) Proliferation was measured using the MTT assay. (**d**) Cell cycle stage was determined by FACS, and the data were analyzed using the ModFit program. Each experiment repeated three times. Data shown are mean ± SD, *P < 0.05 vs. Control.

The neonatal rat CFs were transfected with the BNIP3L and BNIP3L-siRNA adenoviruses. After transfection, cells were collected for the analysis of BNIP3L expression by western blot. The results showed that the adenoviruses of BNIP3L and BNIP3L-siRNA increased and suppressed BNIP3L expression, respectively (Fig. 4a). We detected fibronectin and collagen expression to assess the effect of BNIP3L on ECM accumulation. There was a significant increase and decrease in fibronectin and collagen expression after the transfection of the BNIP3L and BNIP3L-siRNA adenoviruses, respectively (Fig. 4b). Compared with the controls, the MTT assay showed that the over-expression of BNIP3L significantly induced cell proliferation. In contrast, cell proliferation decreased when BNIP3L expression was knocked down by BNIP3L-siRNA (Fig. 4c). Additionally, we found that BNIP3L had the same effect on the cell cycle distribution as the cell proliferation, which was analyzed by flow cytometry (Fig. 4d). These results indicate that BNIP3L significantly regulates ECM protein expression and also affects the cell proliferation of CFs.

**Figure 5:**
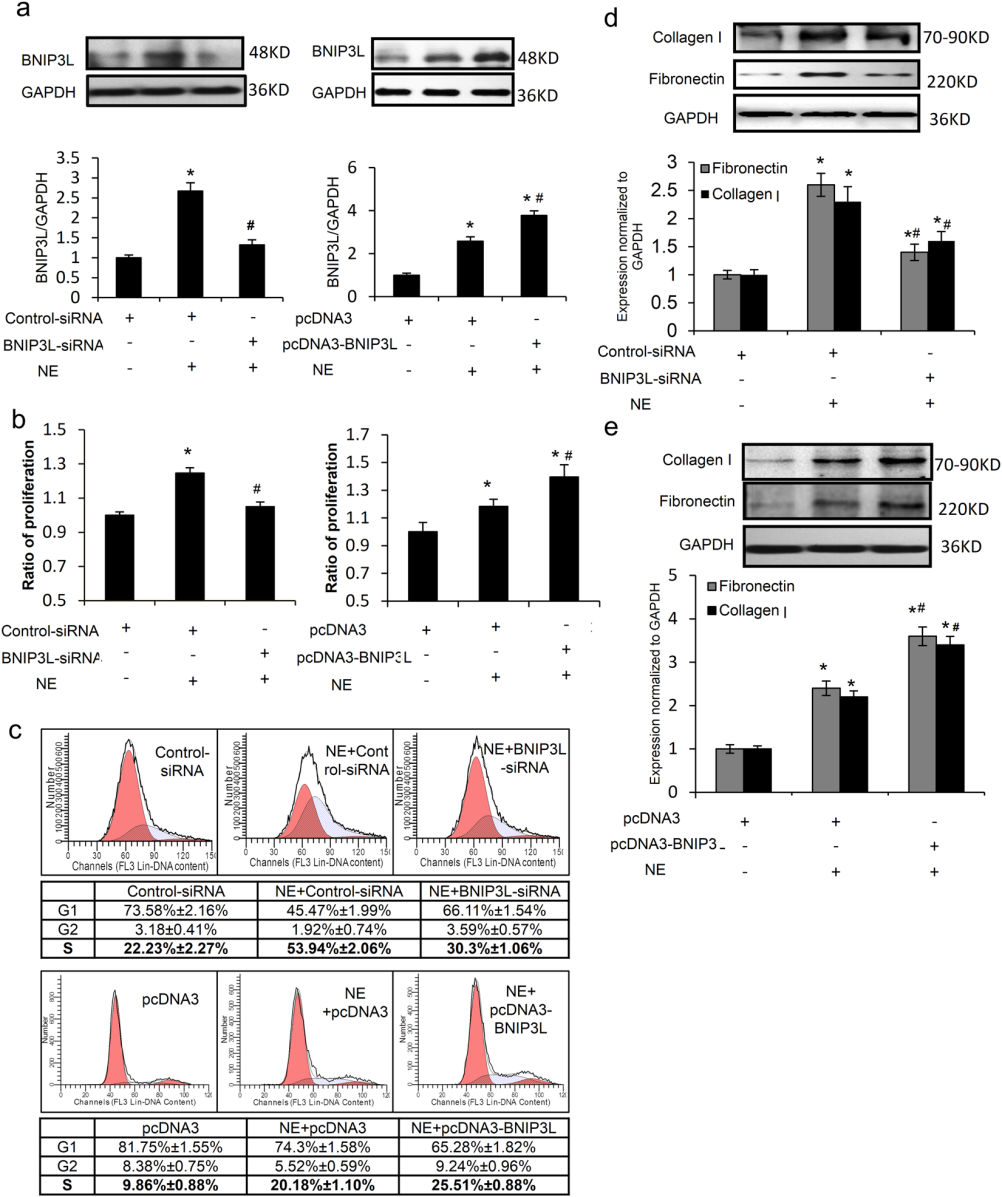
The roles of BNIP3L in NE-treated CFs. Neonatal rat CFs were treated with NE after transfection with the BNIP3L and BNIP3L-siRNA adenoviruses. (**a**) Western blot analysis showing the effects of the transfection on NE-induced BNIP3L expression. (**b**) Proliferation was measured using the MTT assay. (**c**) Cell cycle stage was determined by FACS, and the data were analyzed using the ModFit program. (**d,e**) Western blot analysis showing the effects of BNIP3L on fibronectin and collagen I expression. Each experiment repeated three times. Data shown are mean ± SD, *P < 0.05 vs. Control, ^**#**^P < 0.05 vs. NE.

Chronic exposure to NE is associated with cardiac fibroblast proliferation and collagen secretion, which contributes to the pathophysiology of fibrosis^24^. To evaluate the effects of BNIP3L on NE-induced fibrosis, the transfected neonatal rat CFs were treated with NE. The results showed that the BNIP3L-siRNA and BNIP3L adenoviruses respectively suppressed and increased NE-induced BNIP3L expression (Fig. 5a). Compared with the NE group, flow cytometry analysis showed a significant decrease and increase in the percentages of cells in the DNA synthesis phase after transfection with the BNIP3L-siRNA and BNIP3L adenoviruses, respectively (Fig. 5c), and the same effects were observed for the cell proliferation rate using the MTT assay (Fig. 5b). Additionally, NE had a stimulatory effect on fibronectin and collagen expression, which decreased following the transfection of BNIP3L-siRNA (Fig. 5d). In contrast, the over-expression of BNIP3L aggravated NE-induced fibronectin and collagen expression (Fig. 5e). These results indicate that BNIP3L had significant effects on NE-induced cell proliferation and ECM protein expression.

### BNIP3L promotes cardiac fibrosis through the [Ca^2+^]_i_-TGF-β-Smad2/3 pathway

**Figure 6:**
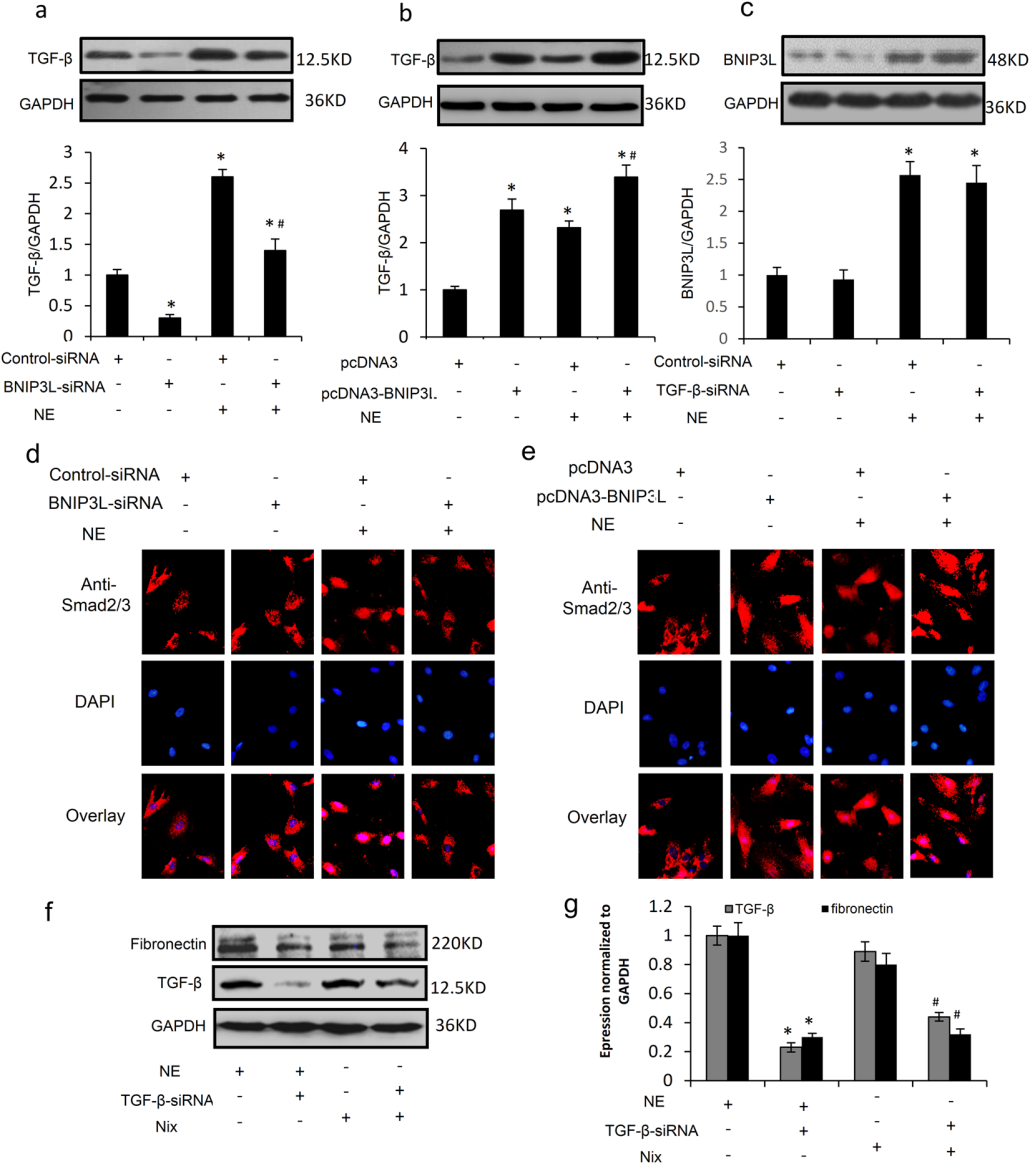
BNIP3L plays an important role in fibrosis via the TGF-β/Smad signaling pathway. Neonatal rat CFs were transfected with the BNIP3L and BNIP3L-siRNA adenoviruses or were treated with NE after the transfection. (**a,b**) Western blot analysis showing the effects of the adenoviruses on the expression of TGF-β (*P < 0.05 vs. Control, ^#^P < 0.05 vs. NE). (**c**) Western blot analysis showing the effects of TGF-β-siRNA on the expression of BNIP3L. (**d,e**) Immunofluorescence images demonstrating the nuclear accumulation of Smad2/3. (**f,i**) Western blot analysis showing the effects of NE, pcDNA3-BNIP3L and TGF-β-siRNA on the TGF-β and fibronectin protein levels (*P < 0.05 vs. NE, ^**#**^P < 0.05 vs. BNIP3L). Each experiment repeated three times.

Transforming growth factor-β (TGF-β) has been long considered to be a key mediator of fibrosis. To investigate whether NE or BNIP3L regulate the classical fibrosis TGF-β/Smad signaling pathway, TGF-β expression was analyzed by western blotting. We found that TGF-β expression decreased following exposure to Ad-BNIP3L-siRNA and increased following exposure to Ad-BNIP3L in neonatal rat CFs (Fig. 6a,b). TGF-β is involved in ECM component synthesis via Smad phosphorylation and nuclear translocation in various cells. BNIP3L overexpression induced the nuclear accumulation of Smad2/3, which is a major intracellular mediator of TGF-β signaling (Fig. 6e). We found that NE is able to activate the TGF-β signaling pathway by increasing TGF-β protein expression and inducing the nuclear accumulation of Smad2/3 (Fig. 6a,b,d,e). The increased TGF-β expression and Smad2/3 nuclear accumulation that are induced by NE were attenuated by Ad-BNIP3L-siRNA but aggravated by Ad-BNIP3L (Fig. 6a,b,d,e). These results indicate that BNIP3L regulates the activation of the TGF-β/Smad signaling pathway. But, Western blot showed no significant change of BNIP3L expression by TGF-β-siRNA (Fig. 6c). To further confirm that BNIP3L is involved in NE-induced fibrosis through the regulation of the TGF-β signaling pathway, the cells were co-transfected with pcDNA3-BNIP3L and TGF-β-siRNA. We found that TGF-β-siRNA attenuated NE- and BNIP3L-induced fibronectin expression (Fig. 6f,g), Taken together, BNIP3L promote fibrosis via the TGF-β-Smad signaling pathway.

**Figure 7:**
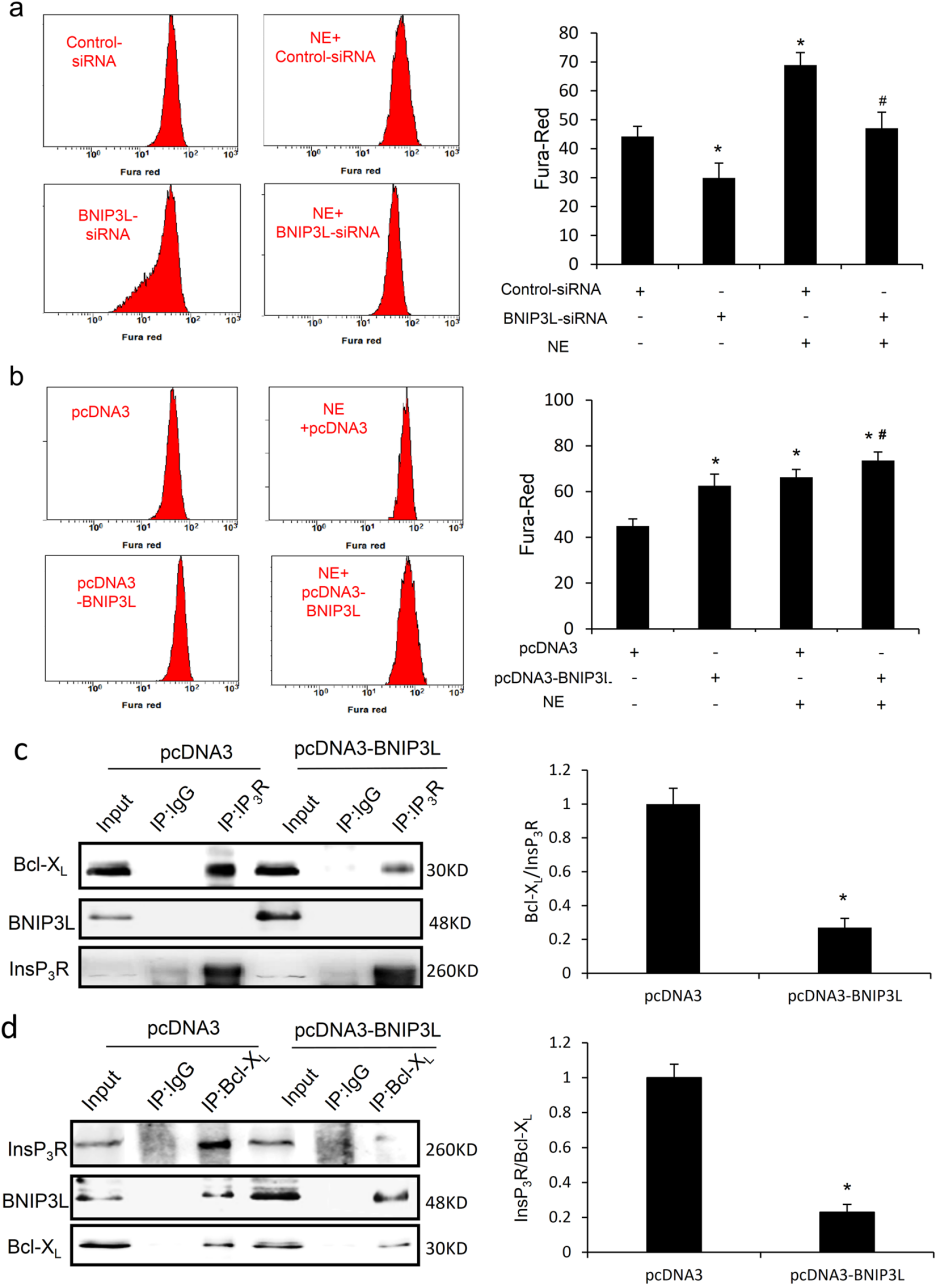
BNIP3L regulates Ca^2+^ in CFs. Neonatal rat CFs were loaded with Fura Red and analyzed by flow cytometry. Data are reported as fluorescence intensities. (**a**) The BNIP3L-siRNA transfected or NE-induced cells were loaded with Fura Red and analyzed as above (*P < 0.05 vs. Control, ^**#**^P < 0.05 vs. NE.). (**b**) The adenoviruses of BNIP3L-transfected or NE-induced cells were loaded with Fura Red and and analyzed as above (*P < 0.05 vs. Control, ^**#**^P < 0.05 vs. NE). (**c**) Co-immunoprecipitation showed the interaction of InsP_3_R with Bcl-X_L_ and BNIP3L when the cells were transfected with pcDNA3-BNIP3L (*P < 0.05 vs. pcDNA3). (**d**) Co-immunoprecipitation showed the interaction of Bcl-X_L_ with InsP_3_R and BNIP3L when the cells were transfected with pcDNA3-BNIP3L (*P < 0.05 vs. pcDNA3). Each experiment repeated three times. Data shown are mean ± SD.

*In vitro* studies have indicated that intracellular Ca^2+^ is an important second messenger in the TGF-β signal transduction pathway. We speculated that BNIP3L regulated the TGF-β/Smad signaling pathway via Ca^2+^ signaling. To determine whether NE or BNIP3L induces changes in intracellular Ca^2+^, the transfected and NE-treated neonatal rat CFs were collected for Ca^2+^ analyses using Fura Red and their intracellular Ca^2+^ levels were determined by flow cytometry. Compared with controls, the Fura Red ratios significantly decreased and increased after transfection with the BNIP3L-siRNA and BNIP3L adenoviruses, respectively (Fig. 7a,b). Treatment with NE caused marked intracellular Ca^2+^ changes. When the neonatal rat CFs were transfected with the adenoviruses expressing BNIP3L (Ad-BNIP3L) and then treated with NE, BNIP3L over-expression aggravated the increase in intracellular Ca^2+^ that is normally induced by NE (Fig. 7b). When BNIP3L expression was inhibited by the specific siRNA (Ad-BNIP3L-siRNA), the NE-induced intracellular Ca^2+^ imbalance was attenuated (Fig. 7a). These data indicate that BNIP3L expression significantly affects the intracellular Ca^2+^.

Inositol 1,4,5-trisphosphate receptor (InsP3R) is a Ca^2+^ release channel. Co-immunoprecipitation showed that overexpression of BNIP3L inhibited the binding of Bcl-X_L_ to InsP_3_R, but no direct interaction was observed between BNIP3L and InsP_3_R (Fig. 7c,d). Moreover, we found that BNIP3L interacted with Bcl-X_L_, which was detected using co-immunoprecipitation (Fig. 7d). These results demonstrated that BNIP3L regulates intracellular Ca^2+^ by interacting with Bcl-X_L_ and inhibiting the binding of Bcl-X_L_ to InsP_3_R.

### BNIP3L is up-regulated through the AR-PKC pathway in pressure overload-induced cardiac fibrosis

**Figure 8:**
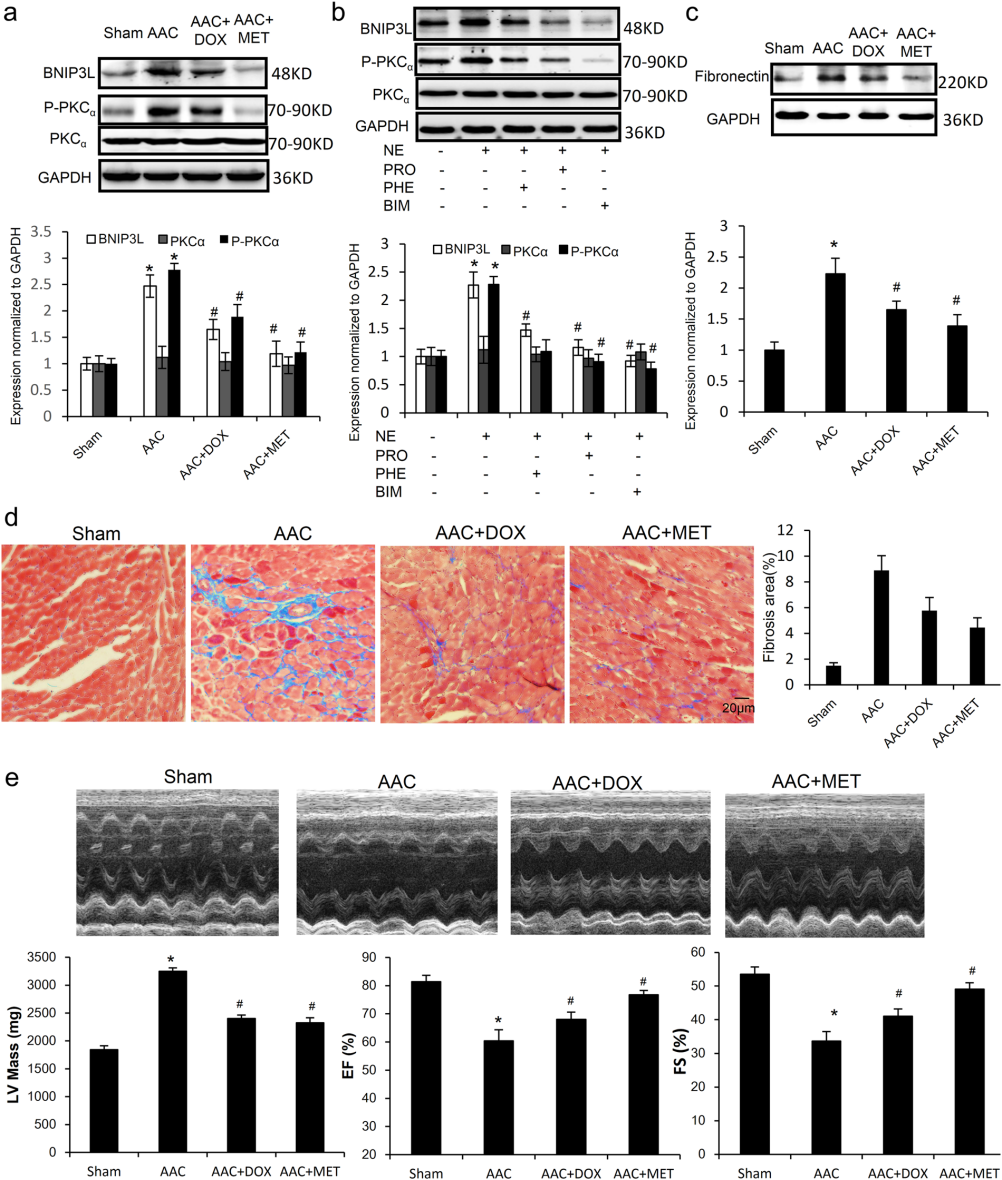
BNIP3L expression was up-regulated by the NE-AR-PKC signaling pathway. (**a**) Western blot analysis showing the effects of doxazosin (DOX) and metoprolol (MET) on AAC-induced BNIP3L. (**b**) Western blot analysis showing the effects of propranolol (PRO), phenoxybenzamine (PHE) and bisindolylmaleimide I (BIM) on NE-induced BNIP3L expression. (**c**) Western blot analysis showing the effects of DOX and MET on AAC-induced fibronectin expression. (**d**) Masson’s trichrome staining showing the effects of DOX and MET on AAC-induced cardiac fibrosis (*P < 0.05 vs. sham, ^**#**^P < 0.05 vs. AAC). (**e**) Echocardiographic assessment showing the effects of DOX and MET on AAC-induced cardiac dysfunction (n = 8, *P < 0.05 vs. sham, ^**#**^P < 0.05 vs. AAC).

To further elucidate the mechanism of up-regulation of BNIP3L in CFs, we attempted to identify the potential regulator of BNIP3L. The AAC rats were treated with the α-adrenoceptor antagonist doxazosin (DOX) or β-adrenoceptor antagonist metoprolol (MET) for 6 weeks following 2 weeks of AAC. We found that doxazosin and metoprolol were able to attenuate AAC-induced BNIP3L expression, which is consistent with PKC activation (Fig. 8a). To verify the role of PKC in CFs in NE-induced BNIP3L expression, the neonatal rat CFs were pretreated with or without the α-adrenoceptor antagonist phenoxybenzamine (PHE), the β-adrenoceptor antagonist propranolol (PRO) and the PKC inhibitor bisindolylmaleimide I (BIM) for 30 min prior to NE (10 µM) treatment. Western blot analysis showed that PHE, PRO and BIM were all capable of attenuating NE-induced BNIP3L expression (Fig. 8b), indicating that pressure overload induces BNIP3L expression via the NE-AR-PKC signaling pathway.

Moreover, the inhibition of BNIP3L expression with doxazosin or metoprolol attenuated AAC-induced fibronectin expression *in vivo* (Fig. 8c). Masson’s trichrome staining on the heart sections demonstrated that the inhibition of BNIP3L expression also attenuated AAC-induced cardiac fibrosis (Fig. 8d). Furthermore, echocardiographic assessment showed that AAC-induced increase in left ventricular (LV) mass and reduction in ejection fraction and fractional shortening were all attenuated by the inhibition of BNIP3L expression, indicating that the inhibition of BNIP3L expression attenuated AAC-induced cardiac dysfunction (Fig. 8e). These results provide evidence that Inhibition of BNIP3L expression could suppress the development of pathological cardiac remodeling through regulating cardiac fibrosis.

## Discussion

Cardiac remodeling after long-term pressure overload results in ventricular dysfunction and heart failure and is considered to be a key determinant of clinical outcome in heart disease^3, 23^. Pathological remodeling involves not only the reactivation of cardiomyocyte death but also CFs proliferation and ECM expression. BNIP3L has been recently shown to be highly transcriptionally up-regulated in Gq-mediated and pressure overload-induced cardiac hypertrophy, including human hypertensive heart disease, and thus, considerable efforts have been made towards elucidating its role in maintaining cardiac function^12, 13, 16^. Previous studies have demonstrated that BNIP3L induced cardiomyocyte death and participates in the process of cardiac remodeling^12, 13^, while it was supposed that apoptotic cell death of cardiomyocytes induced by BNIP3L indirectly leads to increased fibrosis^17^. The direct effects of BNIP3L on fibrosis in CFs remain still unknown.

First, we explored the cellular distribution of BNIP3L in the heart tissue, contributing to a deeper understanding for its function in hypertensive heart disease. Cardiac myocytes and fibroblasts are highly interspersed in the myocardium, with one cardiac myocytes being surrounded by one or more CFs^25^. The fibroblasts are the principal ECM producers, and play central roles in fibrogenesis and myocardial remodeling in heart failure^26^. Besides the increase of BNIP3L in cardiomyocytes during pressure overload-induced heart failure, our findings showed that BNIP3L significantly increased in CFs during pressure overload-induced heart failure *in vivo* and NE-induced fibrosis model *in vitro*. Also, immunolocalization experiments showed that BNIP3L co-localized with DDR2 (a cardiac fibroblast-specific protein). These results provided the first evidence that the location of BNIP3L was not only in cardiac myocytes, but also in CFs during pressure overload-induced heart failure, which indicated that the role of BNIP3L in CFs was necessary for the study about the fibrosis in heart failure.

Previous studies have focused on the role of BNIP3L in cardiomyocytes. Cardiomyocyte-specific ablation or overexpressed of BNIP3L indicated that BNIP3L induces cardiomyocyte death and indirectly leads to increased fibrosis^12, 17^. CFs, the predominant secretory cells producing ECM proteins, are important targets of NE and key mediators of cardiac fibrosis^26^. Here, we found that BNIP3L has significant effects on the cell proliferation rate, cell cycle distribution, collagen and fibronection expression in CFs by regulating BNIP3L expression. Fibronectin is important for the assembly of a collagen matrix *in vitro*. Its continuous presence also supports matrix integrity, both *in vitro* and *in vivo*. It modulates the amount of growth factors in the matric and their release from it^26, 27^ and further regulates cell proliferation and cell cycle progression^28^. Additionally, *in vivo*, the inhibition of BNIP3L expression with DOX or MET attenuated AAC-induced ECM protein expression and collagen production, which further indicates that BNIP3L has pro-fibrotic activities during hypertension. Our study suggested that BNIP3L was responsible for fibroblasts proliferation and ECM production, which provide the first evidence that BNIP3L plays the direct role in CFs in cardiac fibrosis. This could explain that pressure overload-induced cardiac dysfunction incompletely suppressed by cardiomyocyte specific ablation of BNIP3L^17^, which may be due to the upregulation of BNIP3L in CFs to promote fibrosis.

Further, we found a significant increase in the TGF-β level and Smad activation in response to BNIP3L over-expression. Many studies have reported that TGF-β signaling plays an important role in promoting ECM synthesis and fibrosis in the pressure-overloaded mouse heart^29, 30^. The classical Smads, NF-kB and MAPKs signaling pathways have been implicated in the pro-fibrogenic effects of TGF-β^31–33^. TGF-β signals bind to the membrane-bound type I and II TGF-β receptors to activate these receptors, leading to the phosphorylation of the downstream signaling molecules Smad2 and Smad3. Activated Smad2/3 bind to Smad4 and translocate to the nucleus^30, 34^. The Smad2/3/4 complex binds to response elements in the promoter regions of the ECM genes and activates pro-fibrogenic factors by up-regulating gene transcription^30^. The decrease in TGF-β expression and Smad activation were consistent with the knock-down of BNIP3L, which suggests that BNIP3L is an important regulator protein in these processes. According to our observations, NE and BNIP3L induced fibronectin expression, which can be attenuated by TGF-β-siRNA. This raises the possibility that BNIP3L induces fibrosis and exacerbates NE-induced fibrosis through the regulation of the TGF-β/Smad pathway.

How does BNIP3L regulate TGF-β/Smad pathway? Recent studies have demonstrated that Ca^2+^ signaling is essential for fibroblast proliferation, differentiation and ECM-protein production^35^. *In vitro* studies showed Ca^2+^ is an important second messenger of the TGF-β signal transduction pathway^19^. It has been reported that CaMKII inhibitors inhibit the excretion of TGF-β. The calcium channel blocker efonidipine elicits inhibitory effects on TGF-β and Smad2-dependent protein synthesis. We found that the intracellular Ca^2+^ level was affected by the regulation of BNIP3L expression in neonatal rat CFs, which suggest BNIP3L regulates TGF-β/Smad to promote the development of fibrosis by affecting intracellular Ca^2+^ levels. However, the precise manner by which BNIP3L increases the Ca^2+^ level is not yet known. It has been reported that BNIP3L interacts with Bcl-2 and Bcl-X_L_. Bcl-X_L_ is a direct effector of InsP_3_R, resulting in the increased sensitivity of InsP_3_R and enabling Ca^2+^ release from the ER that is more sensitively coupled to extracellular signals. The BH3-only protein tBid inhibits the binding of Bcl-X_L_ to InsP_3_R and antagonizes the effects of Bcl-X_L_ on InsP_3_R channel activity^36^. We found that BNIP3L interacts with Bcl-X_L_ in neonatal rat CFs. Overexpression of BNIP3L inhibited the binding of Bcl-X_L_ to InsP_3_R. These results demonstrated that BNIP3L regulates intracellular Ca^2+^ by interacting with Bcl-X_L_ and inhibiting the binding of Bcl-X_L_ to InsP_3_R to antagonise the effects of Bcl-X_L_ on InsP_3_R channel activity. It has been reported that TRPC3-mediated local Ca^2+^ influx specifically encodes signals to induce cardiac fibrosis during pressure overload induced cardiac remodeling^37, 38^. Also, the TRPC-mediated Ca^2+^ influx is located downstream of InsP_3_R-mediated Ca^2+^ signaling pathway. Future work focusing on the relationship between BNIP3L and TRPC3-mediated Ca^2+^ influx will be required.

The molecular mechanisms of BNIP3L induction have previously been elucidated. BNIP3L is up-regulated by stimuli that are associated with cardiomyocyte hypertrophy, such as phenylephrine application, Gq overexpression and pressure overload, but not isoproterenol, angiotensin II or hypoxia^11^. PKC is a common pathway of phenylephrine application, Gq overexpression and pressure overload and shown to be sufficient for the induction of BNIP3L promoter activity in cultured NRCMs. The signaling events leading to increased BNIP3L expression involves the activation of PKC and the induction of Sp1 and its binding to GC-box motifs in the BNIP3L promoter^11^. As is well known, NE binds to specific adrenoceptor on the cell membrane to induce PKC activation. Our findings extend these data and demonstrate that AAC induces BNIP3L expression via the NE-AR-PKC signaling pathway in CFs.

Taken together, our findings demonstrated that the increase of BNIP3L expression is induced by the PKC signaling pathway, which is activated by the specific binding of NE to α- or β-AR, during pressure overload-induced heart failure. Subsequently, BNIP3L interacts with Bcl-X_L_ and inhibits the binding of Bcl-X_L_ to InsP_3_R, resulting in the increased sensitivity of InsP_3_R and enabling Ca^2+^ release from the ER. The Ca^2+^ signals activate the TGF-β/Smad signaling pathway to promote cardiac fibroblast proliferation and ECM protein expression to participate in cardiac fibrosis. This study suggests a potential link between BNIP3L and cardiac fibrosis in CFs, which may improve our understanding of the molecular mechanisms that are involved in pressure overload-induced heart failure. Further exploration of the unknown functions of BNIP3L and the related signaling mechanisms of fibrosis may provide new insights into future therapeutic targets for heart failure.

## Materials and Methods

### Experimental animals

All animal experimental procedures were conducted in accordance with the Guide for Care and Use of Laboratory Animals (NIH Publication No. 85-23, revised 1996). The protocol was approved by the committee on the Ethics of Animal Experiments of the Beijing Institute of Basic Medical Sciences (Permit Number: 2012-D-3096).

Male Wistar rats weighing 180–200 g (6–8 weeks) were randomly divided into the following groups: control, sham operation and operation. The operation group was subjected to abdominal aortal constriction (AAC) with a 7-gauge syringe needle under anaesthesia with sodium pentobarbital, the sham operation group was treated as previously described, and the control group did not receive any treatment. The rats were sacrificed 2, 4, 6 and 8 weeks after the operations under anaesthesia.

Two weeks after the surgeries, the rats that received the AAC operation were randomized and treated with an α-adrenergic blockade with oral doxazosin (10 mg/kg per day), β-adrenergic blockade with oral metoprolol (20 mg/kg per day), or vehicle for 6 weeks. The rats were sacrificed 8 weeks after the operation under anaesthesia.

### Echocardiography and blood pressure analysis

The rats were anestheNtized by isoflurane and O_2_ inhalation. Echocardiographic measurements were conducted with a high-resolution echocardiography analysis system for small animals (Vevo770, Visual Sonics, Canada). A 2-dimensional short-axis view and M-mode tracings of the left ventricle (LV) were obtained with a 17.5-MHz RMV-716 transducer. Blood pressure measurements were conducted using carotid artery intubation with a MP150 polygraph (BIOPAC, USA).

### Histology

Heart tissues were fixed in 4% paraformaldehyde at 4 °C overnight, embedded in paraffin and sectioned at 5 μm. The sections were stained with hematoxylin/eosin to assess myocardial hypertrophy and with Masson’s trichrome staining to detect fibrosis. Myocardial cell apoptosis analyses were conducted with the *In Situ* Apoptosis Detection Kit IV (Boster, Wuhan). The heart sections were stained with an anti-BNIP3L antibody (Abcam) overnight at 4 °C and then with the appropriate secondary antibody for 1 h at 37 °C. The nuclei were stained with hematoxylin.

### Cell culture

Neonatal rat CFs were isolated from the left ventricles of 1–2-day-old Wistar rats as previously described^39^. The identities of the CFs were confirmed by immunostaining for DDR2.

### Western blot analysis

Immunoblotting analysis was performed as already described elsewhere^40–42^. Briefly, the proteins from hearts or cell lysates were homogenized in RIPA buffer (Sigma-Aldrich) containing phosphatase inhibitor (Phos-STOP, Roche) and protease inhibitor (Complete Mini EDTA-free, Roche). Equal amounts of proteins (50 mg) underwent SDS-polyacrylamide gel electrophoresis (PAGE) and were transferred to polyvinylidene difluoride membranes. Membranes were incubated with primary antibodies (anti-BNIP3L, anti-fibronectin anti-InsP3R: Santa Cruz; anti-TGF-β, anti-smad2/3, anti-Bcl-XL: CST; anti-collagen I: abcam, anti-PKCα and anti-p-PKCα: abcam) and then probed with horseradish peroxidase conjugated secondary antibodies. Blots were visualized with the use of SuperSignal® West Femto Maximum Sensitivity Substrate (Thermo Scientific, USA) and images were captured with an ImageQuant LAS 4000 (GE,USA). The densities of bands were quantified by use of Image-Quant TL software (GE Healthcare).

### MTT assay

Cell viability was determined using the MTT assay (Sigma-Aldrich, St. Louis, MO). The cells were seeded onto a 96-well plate (5,000 cells/well, 100 µl total volume) overnight. Then, cells were treated with the processing mode. After treatment, 10 µl of the MTT stock solution (5 mg/ml) was added to each well, and the cells were incubated at 5% CO_2_ and 37 °C for 4 h. Finally, the media was removed, 100 µl DMSO was added to each well and incubated at room temperature for 15 min. The absorbance was measured at 490 nm with a microplate reader. All experiments were performed in eight wells and repeated three times.

### Flow cytometry

For the apoptosis assays, annexin V staining was performed using the Annexin V-FITC Apoptosis Detection Kit (BD Biosciences, CA, USA) according to the manufacturer’s recommendations. The samples were examined by flow cytometry (FACS Calibur, BD, USA), and the data were analyzed with CELLQuest software (FACS Calibur, BD, USA).

For the cell cycle assays, following incubation, the cells were harvested, washed three times with PBS, centrifuged and fixed with 70% anhydrous ethanol overnight at 4 °C. They were then incubated with RNAase for 30 min and stained with PI for 15 min at 37 °C in the dark. The samples were examined by flow cytometry (FACS Calibur, BD, USA), and the data were analyzed with ModFit LT software (FACS Calibur, BD, USA).

### Adenoviral infection studies

The pcDNA3-BNIP3L plasmid DNNAs were provided by the Center for Pharmacogenomics of Washington University. The siRNAs used to target the BNIP3L gene to suppress BNIP3L expression were synthesized by Genepharma. The recombinant adenoviruses were created by Genechem. NRCFs were infected with the adenoviruses at a titer of 100 PFUs/cell for 48 h at 37 °C as described previously.

### Measurement of intracellular free Ca^2+^ ([Ca^2+^]_i_)

For the Ca^2+^ signal measurements, the cells were harvested, washed with PBS (pH 7.4) three times, incubated with Fura Red (2 μM) for 30 min at 37 °C in the dark and analyzed by flow cytometry (FACS Calibur, BD, USA) at baseline conditions. The concentration of intracellular free Ca^2+^ was recorded as the fluorescence intensity.

### Immunofluorescence

Frozen sections were fixed with pre-cooled acetone. CFs were fixed in 4% paraformaldehyde and permeabilized in 0.2% Triton X-100 in PBS. The sections were stained with anti-BNIP3L and anti-DDR2 antibodies and the CFs were stained with an anti-Smad2/3 antibody overnight at 4 °C and then with the appropriate secondary antibody for 2 h at 37 °C. The nuclei were stained with DAPI.

### Co-immunoprecipitation

The cells were transfected with pcDNA3 or pcDNA3-BNIP3L. At 24 hours after transfection, the cells were collected and were resuspended in RIPA lysis buffer in the presence of a protease inhibitor cocktail. 500 μg of proteins was used for co-immunoprecipitation with the Protein A+G Agarose (Beyotime) according to the manufacturer’s instructions. The eluted proteins were resolved by SDS-PAGE and evaluated using western blot analysis.

### Statistical analysis

The data are expressed as mean as the means ± SEM. Comparisons were performed using Student’s t test or ANOVA as appropriate. A P value of <0.05 denoted statistical significance.

## Electronic supplementary material

Supplementary information

## References

[1] Brown RD, Ambler SK, Mitchell MD, Long CS (2005). The cardiac fibroblast: therapeutic target in myocardial remodeling and failure. Annual review of pharmacology and toxicology.

[2] Nishida M (2011). Roles of heterotrimeric GTP-binding proteins in the progression of heart failure. Journal of pharmacological sciences.

[3] Kehat I (2012). Novel strategies for the treatment of heart failure. Rambam Maimonides medical journal.

[4] Shirani J, Pick R, Roberts WC, Maron BJ (2000). Morphology and significance of the left ventricular collagen network in young patients with hypertrophic cardiomyopathy and sudden cardiac death. Journal of the American College of Cardiology.

[5] Kremneva LV, Abaturova OV (2003). [Molecular and cellular mechanisms of myocardial remodeling in heart failure]. Klinicheskaia meditsina.

[6] Brilla CG, Pick R, Tan LB, Janicki JS, Weber KT (1990). Remodeling of the rat right and left ventricles in experimental hypertension. Circulation research.

[7] Martos R (2007). Diastolic heart failure: evidence of increased myocardial collagen turnover linked to diastolic dysfunction. Circulation.

[8] Dobaczewski, M. & Frangogiannis, N. G. Chemokines and cardiac fibrosis. *Front Biosci* (Schol Ed) **1**, 391–405 (2009). 10.2741/s33 PMC2798729 19482709

[9] Polyakova V (2011). Fibrosis in endstage human heart failure: severe changes in collagen metabolism and MMP/TIMP profiles. International journal of cardiology.

[10] Japp AG, Pettit SJ (2013). Remodeling in heart failure: from the left ventricle to service delivery. Expert review of cardiovascular therapy.

[11] Galvez AS (2006). Distinct pathways regulate proapoptotic Nix and BNip3 in cardiac stress. The Journal of biological chemistry.

[12] Yussman MG (2002). Mitochondrial death protein Nix is induced in cardiac hypertrophy and triggers apoptotic cardiomyopathy. Nature medicine.

[13] Dorn GW, Kirshenbaum LA (2008). Cardiac reanimation: targeting cardiomyocyte death by BNIP3 and NIX/BNIP3L. Oncogene.

[14] Sakata Y, Hoit BD, Liggett SB, Walsh RA, Dorn GW (1998). Decompensation of pressure-overload hypertrophy in G alpha q-overexpressing mice. Circulation.

[15] Dorn GW (2005). Physiologic growth and pathologic genes in cardiac development and cardiomyopathy. Trends in cardiovascular medicine.

[16] Syed F (2004). Physiological growth synergizes with pathological genes in experimental cardiomyopathy. Circulation research.

[17] Diwan A (2008). Nix-mediated apoptosis links myocardial fibrosis, cardiac remodeling, and hypertrophy decompensation. Circulation.

[18] Du J (2010). TRPM7-mediated Ca2+ signals confer fibrogenesis in human atrial fibrillation. Circulation research.

[19] Nesti LJ (2007). TGF-beta1 calcium signaling in osteoblasts. Journal of cellular biochemistry.

[20] Kamato D (2013). Transforming growth factor-beta signalling: role and consequences of Smad linker region phosphorylation. Cellular signalling.

[21] Kong P, Christia P, Frangogiannis NG (2014). The pathogenesis of cardiac fibrosis. Cellular and molecular life sciences: CMLS.

[22] Manolis, A. J., Poulimenos, L. E., Kallistratos, M. S., Gavras, I. & Gavras, H. Sympathetic overactivity in hypertension and cardiovascular disease. *Current vascular pharmacology* (2013). 10.2174/15701611113119990140 23905597

[23] Levy D (2002). Long-term trends in the incidence of and survival with heart failure. The New England journal of medicine.

[24] Leicht M, Greipel N, Zimmer H (2000). Comitogenic effect of catecholamines on rat cardiac fibroblasts in culture. Cardiovascular research.

[25] Zhang P, Su J, Mende U (2012). Cross talk between cardiac myocytes and fibroblasts: from multiscale investigative approaches to mechanisms and functional consequences. American journal of physiology. Heart and circulatory physiology.

[26] Eghbali M (1992). Cardiac fibroblasts: function, regulation of gene expression, and phenotypic modulation. Basic research in cardiology.

[27] Velling T, Risteli J, Wennerberg K, Mosher DF, Johansson S (2002). Polymerization of type I and III collagens is dependent on fibronectin and enhanced by integrins alpha 11beta 1 and alpha 2beta 1. The Journal of biological chemistry.

[28] Manabe R, Oh-e N, Sekiguchi K (1999). Alternatively spliced EDA segment regulates fibronectin-dependent cell cycle progression and mitogenic signal transduction. The Journal of biological chemistry.

[29] Lucas JA (2010). Inhibition of transforming growth factor-beta signaling induces left ventricular dilation and dysfunction in the pressure-overloaded heart. American journal of physiology. Heart and circulatory physiology.

[30] Gong K (2011). Transforming growth factor-beta inhibits myocardial PPARgamma expression in pressure overload-induced cardiac fibrosis and remodeling in mice. Journal of hypertension.

[31] Leivonen SK, Hakkinen L, Liu D, Kahari VM (2005). Smad3 and extracellular signal-regulated kinase 1/2 coordinately mediate transforming growth factor-beta-induced expression of connective tissue growth factor in human fibroblasts. The Journal of investigative dermatology.

[32] Luedde T, Schwabe RF (2011). NF-kappaB in the liver–linking injury, fibrosis and hepatocellular carcinoma. *Nature reviews*. Gastroenterology & hepatology.

[33] Jia D (2013). Up-regulation of RACK1 by TGF-beta1 promotes hepatic fibrosis in mice. PloS one.

[34] Shi Y, Massague J (2003). Mechanisms of TGF-beta signaling from cell membrane to the nucleus. Cell.

[35] Yue Z, Zhang Y, Xie J, Jiang J, Yue L (2013). Transient receptor potential (TRP) channels and cardiac fibrosis. Current topics in medicinal chemistry.

[36] White C (2005). The endoplasmic reticulum gateway to apoptosis by Bcl-X(L) modulation of the InsP3R. Nature cell biology.

[37] Kitajima N (2016). TRPC3 positively regulates reactive oxygen species driving maladaptive cardiac remodeling. Scientific reports.

[38] Numaga-Tomita T (2016). TRPC3-GEF-H1 axis mediates pressure overload-induced cardiac fibrosis. Scientific reports.

[39] Tsuruda T (1999). An autocrine or a paracrine role of adrenomedullin in modulating cardiac fibroblast growth. Cardiovascular research.

[40] Maruyama S (2016). Follistatin-like 1 promotes cardiac fibroblast activation and protects the heart from rupture. EMBO molecular medicine.

[41] Li L (2011). Angiotensin II increases periostin expression via Ras/p38 MAPK/CREB and ERK1/2/TGF-beta1 pathways in cardiac fibroblasts. Cardiovascular research.

[42] Qu X (2017). MIAT Is a Pro-fibrotic Long Non-coding RNA Governing Cardiac Fibrosis in Post-infarct Myocardium. Scientific reports.

