# Supplementary material for: BNIP3L promotes cardiac fibrosis in cardiac fibroblasts through [Ca2+]i-TGF-β-Smad2/3 pathway
Source: Sci Rep. 2017 May 15;7:1906. doi: 10.1038/s41598-017-01936-5 (PMC5432493; doi:10.1038/s41598-017-01936-5)
Supplement: Supplementary file 1 — Supplementary information [file 41598_2017_1936_MOESM1_ESM.pdf]

BNIP3L promotes cardiac fibrosis in cardiac fibroblasts through $[Ca^{2+}]$i-TGF-β-Smad2/3 pathway

Weili Liu[1,2], Xinxing Wang[1,2], Zhusong Mei[2], Jingbo Gong[2], lishuang Huang[2], Xiujie Gao[1,2], Yun Zhao[2], Jing Ma[2], Lingjia Qian[2]*

1. Tianjin Institute of Health and Environmental Medicine, No.1 Da Li Road, Heping District,Tianjin 300050, China

2 .Beijing Institute of Basic Medical Sciences, No. 27 Taiping Road, Haidian District, Beijing 100850, China

Supplementary Figure. 1

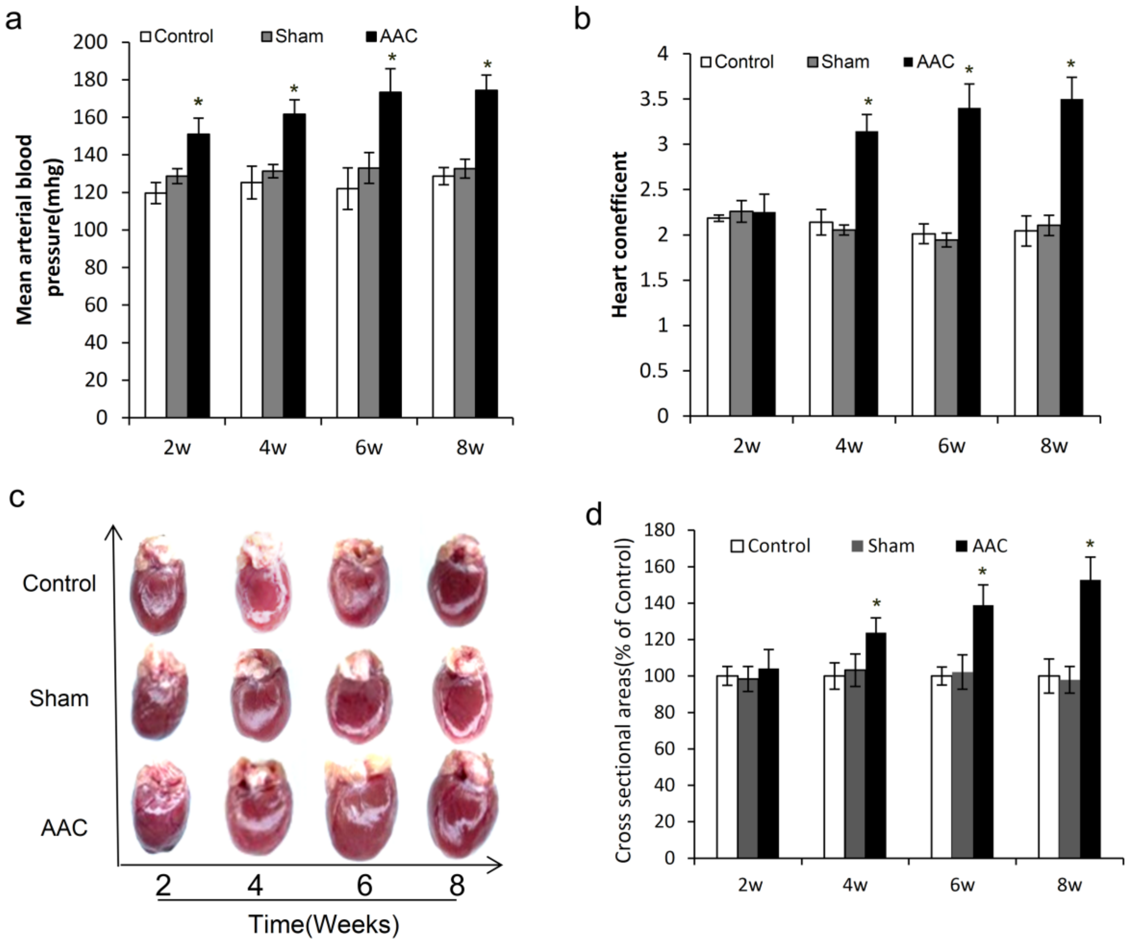

Supplementary Figure. 1 Abdominal aortic constriction-induced hypertension, myocardial hypertrophy and apoptosis. Rats were subjected to abdominal aortic constriction (AAC) surgery. **(a)** Mean arterial blood pressure (*P≤0.05 vs. control and sham, n=8). **(b)** Heart weight/body weight ratios (*P≤0.05 vs. control and sham, n=8). **(c)** Gross morphologies of the whole hearts. **(d)** Cross sectional areas of cardiomyocytes.

Supplementary Figure. 2

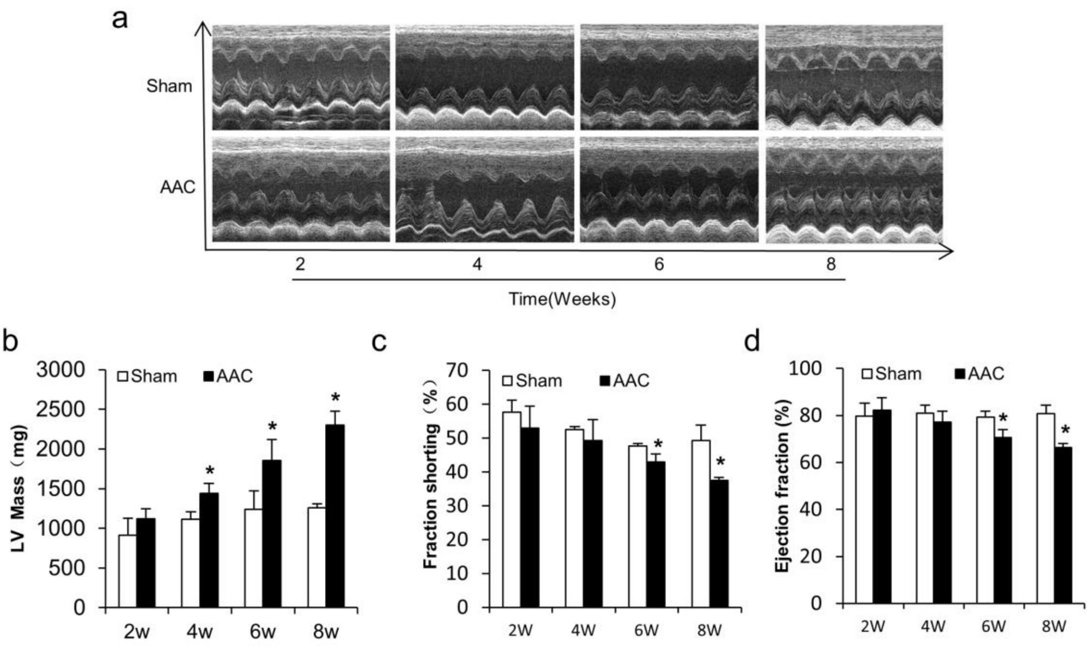

Supplementary Figure. 2 Echocardiographic parameters of the rats with pressure overload. Rats were subjected to abdominal aortic constriction (AAC) surgery. The results are shown at 2, 4, 6 and 8 weeks after the AAC or sham operations. **(a)** Representative left ventricular (LV) M-mode echocardiograms in short axis. **(b)** Echocardiographic left ventricular mass. **(c)** Echocardiographic left ventricular fractional shortening (%FS). **(d)** Echocardiographic left ventricular ejection fraction (%EF). Data shown are mean±SD,*P≤0.05 vs. sham, n=8.

Supplementary Figure. 3

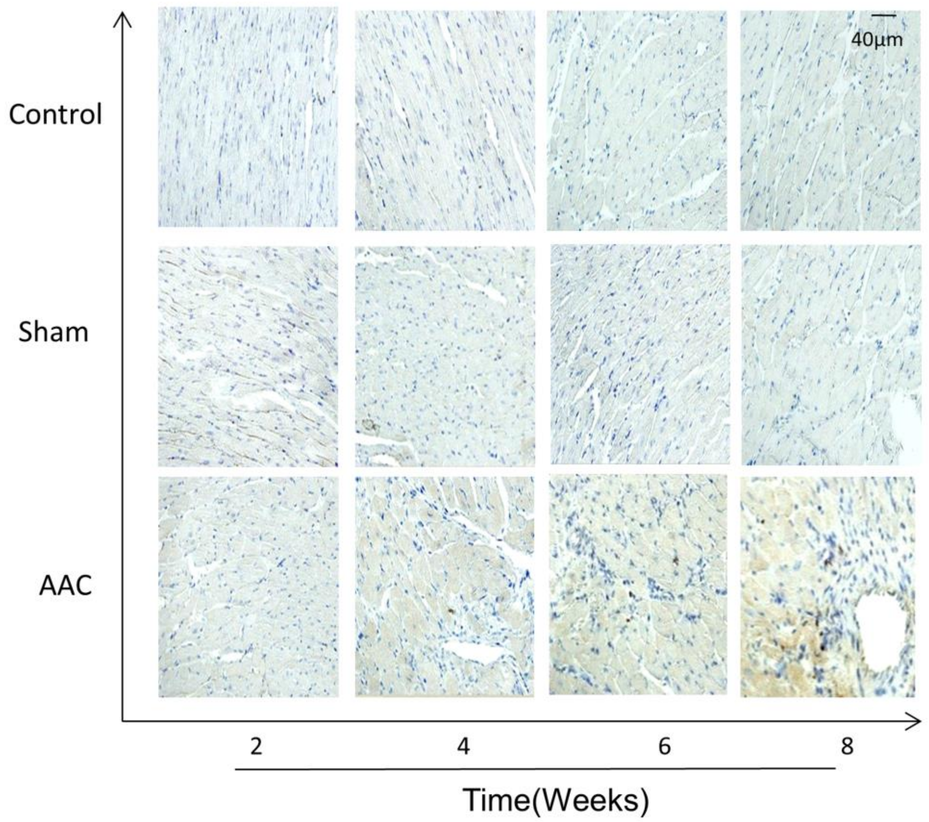

Supplementary Figure. 3 TUNEL staining showing the effects of pressure overload on cell apoptosis.

Supplementary Figure. 4

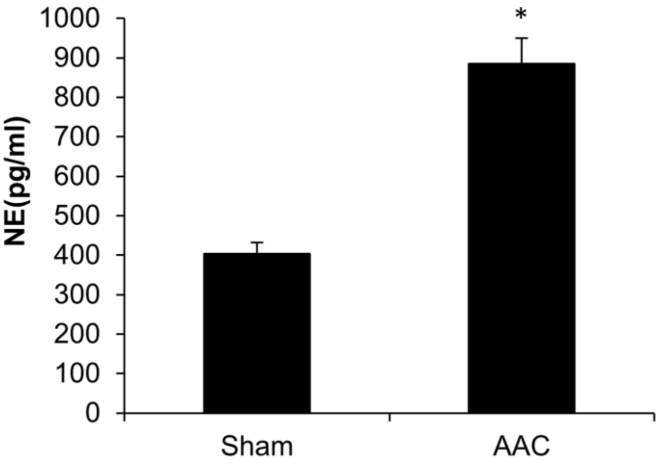

Supplementary Figure. 4 The plasma NE concentrations in rats subjected to AAC. Rats were sacrificed 8 weeks after AAC. An ELISA kit (IBL, Germany) was used to detect the NE levels in the plasma. (*P<0.05 vs. sham, n=8).

Supplementary Figure. 5

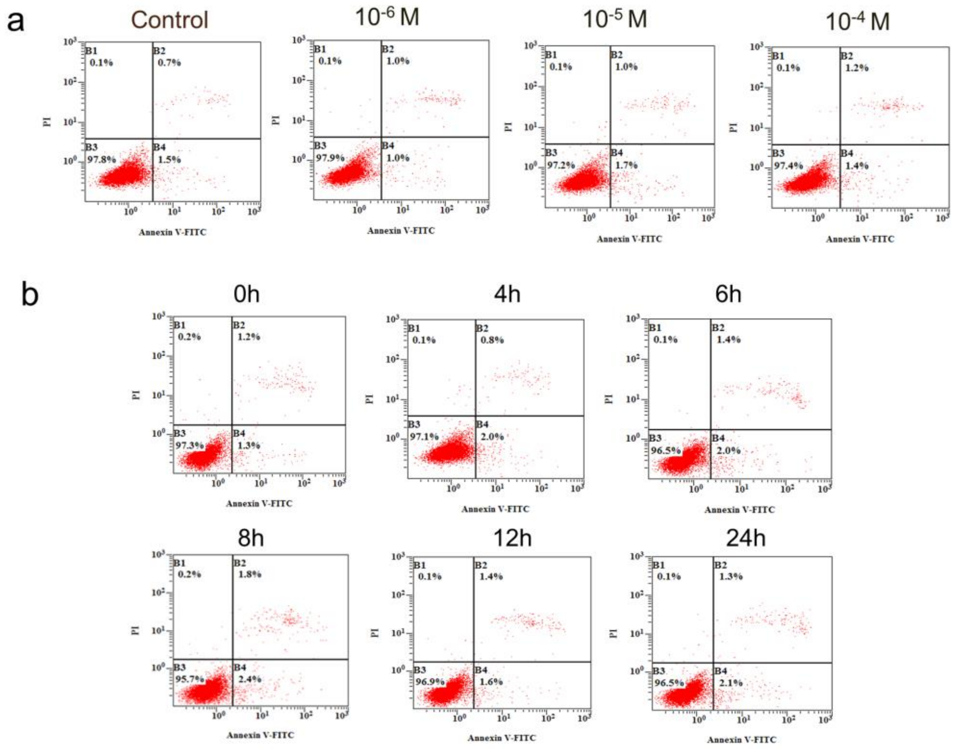

Supplementary Figure. 5 The effects of NE on cardiac fibroblast apoptosis. The neonatal rat cardiac fibroblasts were treated with different concentrations of NE for the indicated times. **(a and b)** The cells were stained with propidium iodide and annexin V for flow cytometry and analyzed for apoptosis using CELLQuest software. Each experiment repeated three times.

Supplementary Figure. 6

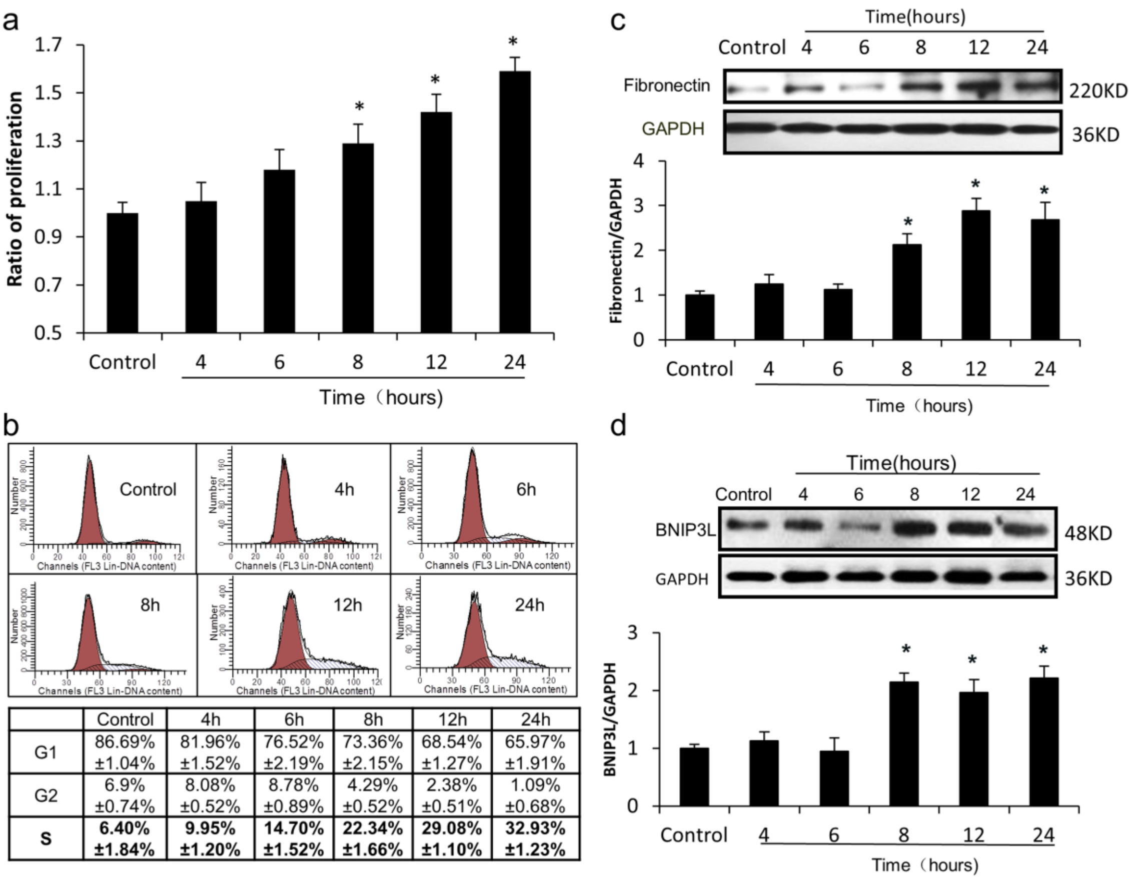

	Control	4h	6h	8h	12h	24h
G1	86.69% ±1.04%	81.96% ±1.52%	76.52% ±2.19%	73.36% ±2.15%	68.54% ±1.27%	65.97% ±1.91%
G2	6.9% ±0.74%	8.08% ±0.52%	8.78% ±0.89%	4.29% ±0.52%	2.38% ±0.51%	1.09% ±0.68%
**S**	**6.40% ±1.84%**	**9.95% ±1.20%**	**14.70% ±1.52%**	**22.34% ±1.66%**	**29.08% ±1.10%**	**32.93% ±1.23%**

Supplementary Figure. 6 The effects of NE on cell fibrosis and BNIP3L expression in cardiac fibroblasts. CFs were treated with 10^{-5} mol/l NE for the indicated times. **(a)** Proliferation was measured using the MTT assay (*P<0.05 vs. control). **(b)** Cells were stained with PI and examined by FACS. The data were analyzed using the ModFit program. **(c)** Western blot analysis showing NE- induced fibronectin expression. **(d)** Western blot analysis showing NE- induced BNIP3L expression. Each experiment repeated three times. Data shown are mean±SD, *P<0.05 vs. Control.
